# Structure of the thermo-sensitive TRP channel TRP1 from the alga *Chlamydomonas reinhardtii*

**DOI:** 10.1038/s41467-019-12121-9

**Published:** 2019-09-13

**Authors:** Luke L. McGoldrick, Appu K. Singh, Lusine Demirkhanyan, Ting-Yu Lin, Ryan G. Casner, Eleonora Zakharian, Alexander I. Sobolevsky

**Affiliations:** 1 Department of Biochemistry and Molecular Biophysics, Columbia University, 650 West 168th Street, New York, NY 10032 USA; 2 Integrated Program in Cellular, Molecular and Biomedical Studies, Columbia University, 650 West 168th Street, New York, NY 10032 USA; 3 Department of Cancer Biology & Pharmacology, The University of Illinois College of Medicine at Peoria, One Illini Drive, Peoria, IL 61605 USA

**Keywords:** Cryoelectron microscopy, Ion transport

## Abstract

Algae produce the largest amount of oxygen on earth and are invaluable for human nutrition and biomedicine, as well as for the chemical industry, energy production and agriculture. The mechanisms by which algae can detect and respond to changes in their environments can rely on membrane receptors, including TRP ion channels. Here we present a 3.5-Å resolution cryo-EM structure of the transient receptor potential (TRP) channel crTRP1 from the alga *Chlamydomonas reinhardtii* that opens in response to increased temperature and is positively regulated by the membrane lipid PIP_2_. The structure of crTRP1 significantly deviates from the structures of other TRP channels and has a unique 2-fold symmetrical rose-shape architecture with elbow domains and ankyrin repeat domains submerged and dipping into the membrane, respectively. Our study provides a structure of a TRP channel from a micro-organism and a structural framework for better understanding algae biology and TRP channel evolution.

## Abstract (web summary)

Transient receptor potential (TRP) channels are ubiquitous occurring cation-selective sensory ion channels that respond to various stimuli. Here the authors characterize crTRP1 from the alga *Chlamydomonas reinhardtii*, present its 3.5 Å cryo-EM structure and show that crTRP1 opens in response to increased temperature and is positively regulated by the membrane lipid PIP_2_.

## Introduction

Algae contribute to marine food chains (phytoplankton), form symbiotic relationships with other organisms, such as sea sponges, coral reef-forming invertebrates, or lichens, are considered ecological indicators and can be used for pollution control, detoxication, and water filtration (algae scrubber)^1^. Algae are sources of food rich in vitamins, minerals, and unsaturated fatty acids^2,3^. They are also sources of agar and alginates for the food and biomedical industries, as well as of polymers and natural pigments for the chemical industry^3^. In agriculture, algae can be used as fertilizers^4^. Finally, due to an enormous potential to produce more biomass per unit area than any other organism, algae hold promise to become a future biofuel^5^.

Algae are guided by multiple sensory cues, many of which are detected by membrane ion channels. Transient receptor potential (TRP) channels are ubiquitous cation-selective sensory ion channels that respond to numerous physical and chemical stimuli and are often considered polymodal signal integrators^6–8^. Several TRP channels have been identified and partially characterized in different species of algae^9,10^. Correspondingly, a toolkit of mammalian TRP channel agonists and antagonists has been shown to positively and negatively regulate cellular calcium uptake in *Ulva compressa*^11^ and *Ectocarpus siliculosus*^12^. Multiple TRP channels have been identified in the fresh water, biflagellate alga *Chlamydomonas reinhardtii*^9,10^. Similar to its mammalian counterpart expressed in the primary cilia of the kidney, the PKD2 channel of *C. reinhardtii* was shown to localize to the cell’s flagellar^13^. The depletion of PKD2 resulted in altered phosphorylation of a cGMP-dependent protein kinase and reduced mating efficiency^13^. Suppressed expression of TRP11, another TRP channel localized to the flagellar, eliminated the *C. reinhardtii* avoiding reaction, a reversal in swimming direction as a response to collision^10^.

Another previously characterized TRP channel from *C. reinhardtii*^9^, crTRP1, has not been identified in flagella but exhibited elevated expression in response to deflagellation^10^. crTRP1 is inhibited by classical TRP channel antagonists BCTC, La^3+^ and Ruthenium Red, is activated by heat and become less active upon phosphatidylinositol-4,5-bisphosphate (PIP_2_) depletion^9^. Although there is a wealth of TRP channel structural information representing multicellular organisms, such information is missing from microbes, limiting our understanding of the structural and functional diversity of this superfamily of ion channels. Therefore, we have decided to structurally and functionally characterize this unique representative.

In this study, we present cryo-electron microscopy (cryo-EM) structures of crTRP1. The crTRP1 architecture is unique; it exhibits overall C2 symmetry and its ankyrin repeat domain (ARD) appears to bend towards and interact with the lipid bilayer. In agreement with a previous study, we found that crTRP1 requires both heat and the lipid PIP_2_ for its activation.

## Results

### Structural and functional characterization

**Fig. 1:**
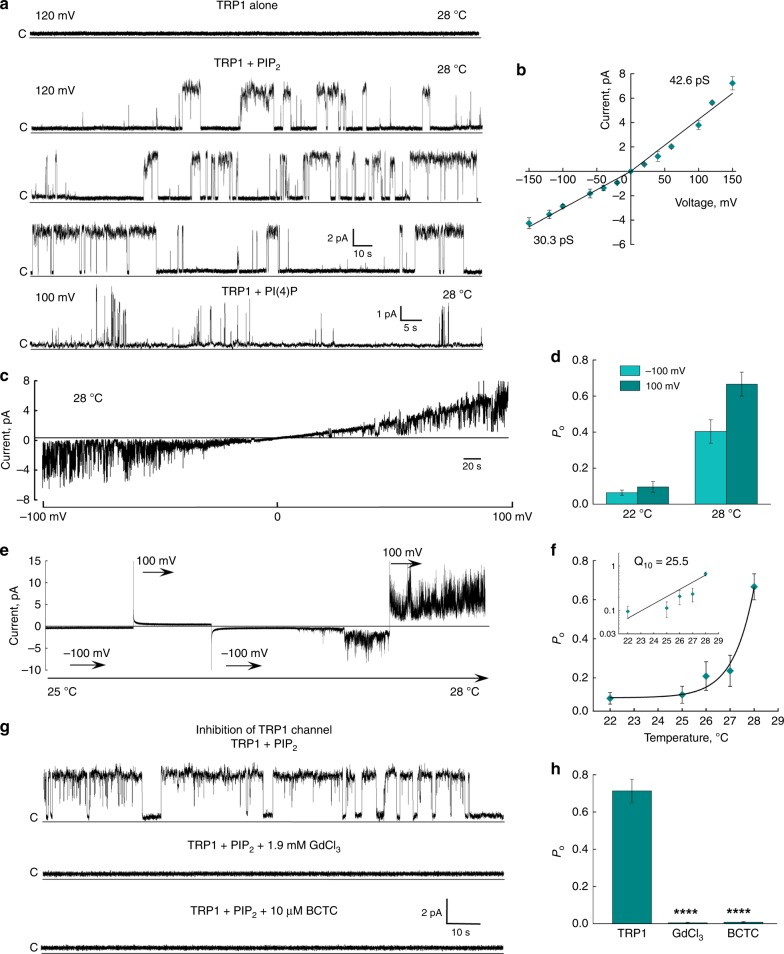
Functional characterization of crTRP1. **a** Representative single-channel recordings of crTRP1 currents obtained in the absence of phosphoinositides or in the presence of 2.5 µM PIP_2_ at 28 °C and +120 mV (*n* = 5; the number of events analyzed (NE) = 2538) or PI(4)P at 28 °C and +100 mV (*n* = 3; NE = 650). **b** Current–voltage (*I*–*V*) relationship for crTRP1 activity evoked by 2.5 µM PIP_2_ at 28 °C. The data represent 15 independent experiments, NE = 114,629. **c** Representative single-channel traces obtained from voltage ramps of −100 to +100 mV (representative of *n* = 8). **d** crTRP1 channel open probability (*P*_o_) obtained at 22 and 28 °C, and two different voltages, −100 and +100 mV (*n* = 18; NE = 192,809). **e** Representative crTRP1 current traces obtained in the presence of 2.5 µM PIP_2_ in response to a temperature increase from 22 to 28 °C, at −100 and +100 mV (representative of *n* = 15). **f** Temperature dependence of *P*_o_ in the presence of 2.5 µM PIP_2_, obtained at +100 mV (*n* = 12; NE = 67,673). Insert, *Q*_10_ was estimated from the regression slope of temperature dependent changes in *P*_o_, *Q*_10_ = 25.5 ± 1.2. **g** Representative crTRP1 current traces recorded at 28 °C, +120 mV, and 2.5 µM PIP_2_ (upper traces), in the presence of 1.9 mM GdCl_3_ (middle traces), or 10 µM BCTC (lower traces). **h** Summary of crTRP1 inhibition by 1.9 mM GdCl_3_ or 10 µM BCTC (*n* = 3 for each inhibitor, NE = 8,560; difference in *P*_o_ values was calculated using One-way ANOVA, *p* = 1.6 × 10^−5^ for the control vs. GdCl_3_, and *p* = 1.7 × 10^−5^ for the control vs. BCTC). All data are presented as mean ± SEM. Source data are provided as a Source Data file

We heterologously expressed the full-length crTRP1 in HEK 293S cells and purified it in the detergent glycol-diosgenin (GDN) or lauryl maltose neopentyl glycol (LMNG) (Methods). Purified crTRP1 was reconstituted in planar lipid bilayers and its function was tested in various lipids and at different temperatures (Fig. 1). We observed no channel openings when crTRP1 was incorporated into the bilayers in the absence of phosphoinositides. Addition of PIP_2_ readily evoked channel openings. Similar openings were also observed in the presence of phosphatidylinositol-4-phosphate PI(4)P (Fig. 1a), suggesting that the channel activity is regulated by phosphoinositides. Like other TRP channels, crTRP1 demonstrated outwardly rectifying currents, where rectification is a function of both differences in single-channel conductance and open probability (*P*_o_). Consistent with previous cellular recordings^9^, crTRP1 activation was strongly temperature dependent (Fig. 1c–f). Fitting of the *P*_o_ temperature dependence (Methods, Fig. 1f) yielded a high temperature coefficient, *Q*_10_ = 25 ± 1 (*n* = 15), which closely resembles the *Q*_10_ for the temperature sensitive mammalian TRP channels TRPM8^14^ and TRPV1^15,16^. We also examined the effect of TRP1 inhibitors and found that both gadolinium and *N*-(4-tertiarybutylphenyl)-4-(3-cholorphyridin-2-yl)tetrahydropyrazine-1(2 H)-carbox-amide (BCTC) strongly suppressed crTRP1-mediated currents (Fig. 1g, h), as was found previously^9^.

To characterize crTRP1 structurally, the protein was subjected to single-particle cryo-EM. The final reconstruction of crTRP1 in GDN exhibited an overall resolution of 3.53 Å, with higher resolution for the molecule’s core (Supplementary Table 1, Supplementary Fig. 1). In an attempt to better mimic a native membrane environment, we also generated a crTRP1 reconstruction in nanodisc (Supplementary Table 1, Supplementary Fig. 2). Although the crTRP1 reconstruction in nanodisc exhibited slightly higher resolution (3.45 Å) and a somewhat better resolved pore-loop (P-loop) region (Supplementary Fig. 2 g, h), the resulting structure was nearly identical to the structure in GDN (Supplementary Fig. 3). The high quality of our cryo-EM maps (Supplementary Fig. 4) allowed de novo building of 901 residue-long crTRP1 tetramer subunits. We could not resolve or build 16 N-terminal and 27 C-terminal residues, 43 residues in the linker domain and 28 residues in the pore domain, presumably because of higher flexibility in these regions.

### crTRP1 architecture

**Fig. 2:**
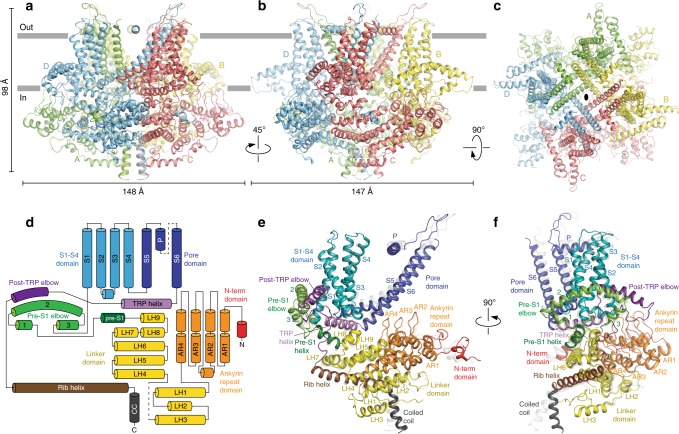
Architecture and domain organization of crTRP1. **a**–**c** Two side (**a**, **b**) and top (**c**) views of the crTRP1 tetramer, with each subunit shown in a different color. **d** Domain organization diagram of a crTRP1 subunit. **e**, **f** Two views of a crTRP1 subunit, with domains colored as in **d**

Unlike structures of the majority of mammalian TRP channels^15,17–25^, the rose-shaped structure of the crTRP1 homotetramer has twofold, rather than fourfold rotational symmetry (Fig. 2a–c). The central transmembrane domain of each crTRP1 subunit includes the S1–S4 and pore domains in the canonical domain-swapped arrangement, followed by an amphipathic TRP helix that runs parallel to the membrane (Fig. 2d–f). The periphery of the transmembrane domain includes hydrophobic pre-S1 and post-TRP elbows that both reenter the membrane from its intracellular side and reach toward the middle of the bilayer. Similar elbow domains have been observed in NOMPC^23^, TRPC3 (refs. ^26,27^), TRPC4 (ref. ^25^), TRPC6 (ref. ^26^), TRPM2 (refs. ^19,24^), TRPM4 (refs. ^20^), TRPM7 (ref. ^25^), and TRPM8 (refs. ^21,28^).

**Fig. 3:**
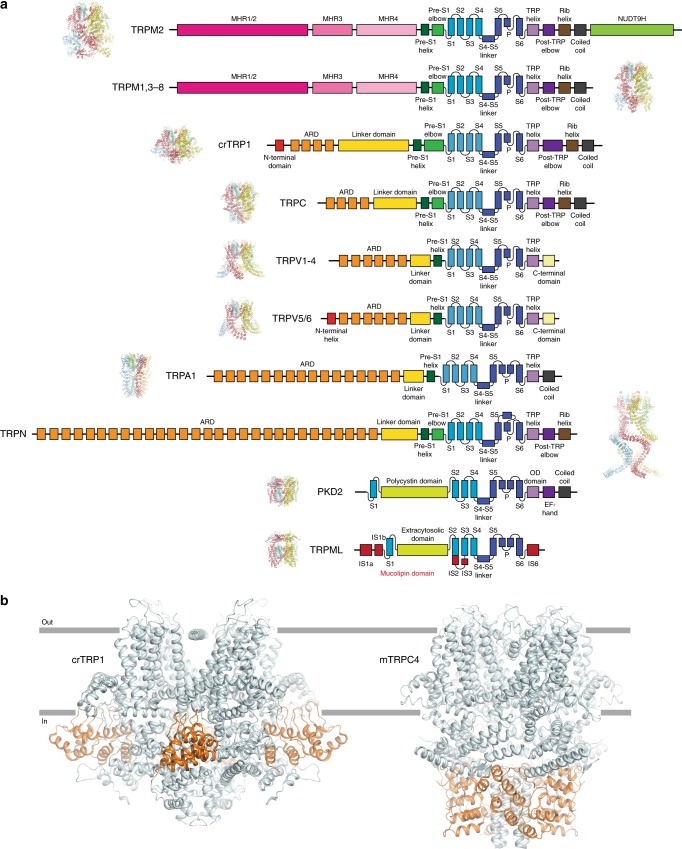
Comparison of the domain organizations of different TRP channels. **a** Linear domain topologies for different TRP channel subtypes aligned relative to the transmembrane domain with structural architectures illustrated by zebrafish TRPM2 (PDB ID: 6DRJ), human TRPM4 (PDB ID: 6BQV), alga crTRP1, mouse TRPC4 (PDB ID: 5Z96), mouse TRPV3 (PDB ID: 6DVY), human TRPV6 (PDB ID: 6B08), human TRPA1 (PDB ID: 3J9P), drosophila TRPN (PDB ID: 5VKQ), human PKD2 (PDB ID: 5T4D), and marmoset TRPML3 (PDB ID: 5W3S). **b** Structures of crTRP1 (left) and mouse TRPC4 (right, PDB ID: 5Z96) viewed parallel to membrane and illustrating different positioning of the ARDs (orange) relative to membrane (gray horizontal lines)

The large intracellular domain of crTRP1 adopts a unique fold when compared to other TRP channels (Fig. 3a). The distal N terminus (residues 17–50) interacts extensively with the intracellular domain of the neighboring subunit, suggesting its role in stabilizing the channel tetrameric assembly. Following the N-terminal domain is the ARD that is comprised of four ankyrin repeats whose fingers, surprisingly, extend towards and dip into the plasma membrane, a feature unique to crTRP1 that has not been observed in any member of the TRP channel family. This feature defines the distinct crTRP1 architecture and is especially striking when compared to the architecture of TRPC channels, which have otherwise similar domain compositions (Fig. 3b). Similar interactions with the membrane were proposed for the ankyrin-G family proteins^29^, which regulate the function of various ion channels and transporters^30^. A linker domain comprised of nine alpha helices connects the ARD with the TM domain, similar to the TRPC channels^25,26^. Following the post-TRP elbow is a rib helix, a 33 residue-long α-helical segment that runs parallel to TRP helix, and then a 12 residue-long α-helix that, along with similar helices of other subunits, forms a coiled coil, perpendicular to the membrane and in line with the ion conduction pathway. Similar structural elements were observed in TRPC3 (refs. ^26,27^), TRPC4 (ref. ^25^), TRPC6 (ref. ^26^), TRPM2 (refs. ^19,24^), TRPM4 (ref. ^20^), TRPM7 (ref. ^25^), and TRPM8 (ref. ^21^).

**Fig. 4:**
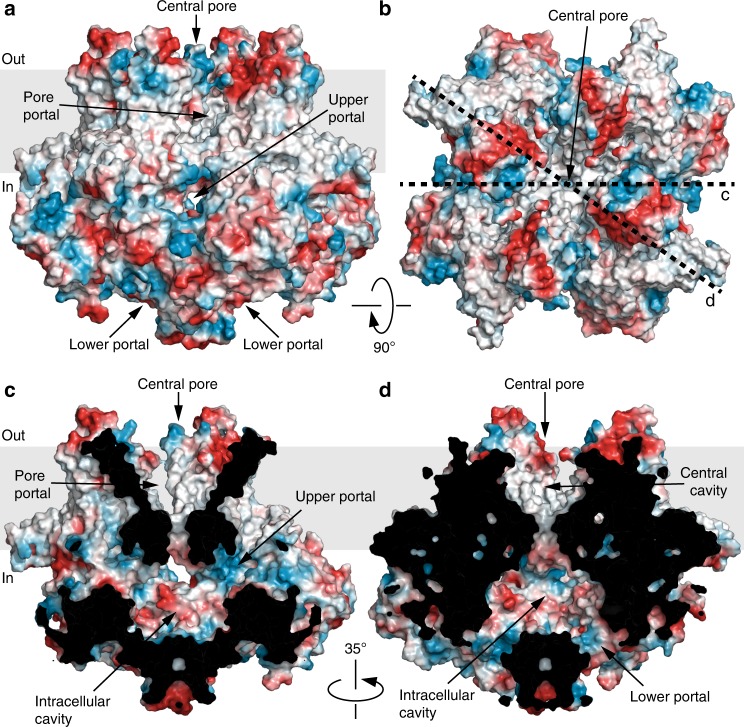
Surface electrostatics, central pore and portals. **a**, **b** Side (**a**) and top (**b**) views of crTRP1 structure in surface representation, colored by electrostatic potential. **c**, **d** Central slices of crTRP1 surface through the upper (**c**) and the lower (**d**) portals in the directions indicated by the dotted lines in **b**

Because of tight packing of the coiled coil, there is no straight continuous pathway for permeant ions through crTRP1 (Fig. 4). Instead, the central transmembrane pore opens into a wide intracellular cavity that connects to the cytoplasm via two sets of portals, four upper portals formed by the N-terminal domain and ARD of one subunit and the linker domain of the neighboring subunit, and four lower portals, each between two neighboring rib helices and a linker domain. The central pore includes a large central cavity, which has four pore portals that run perpendicular to the pore axis towards the membrane. The pore portals are unlikely to conduct ions, but similar to voltage-gated sodium channels^31^, may provide access to the pore for small hydrophobic molecules or lipids.

### Lipid-like densities

**Fig. 5:**
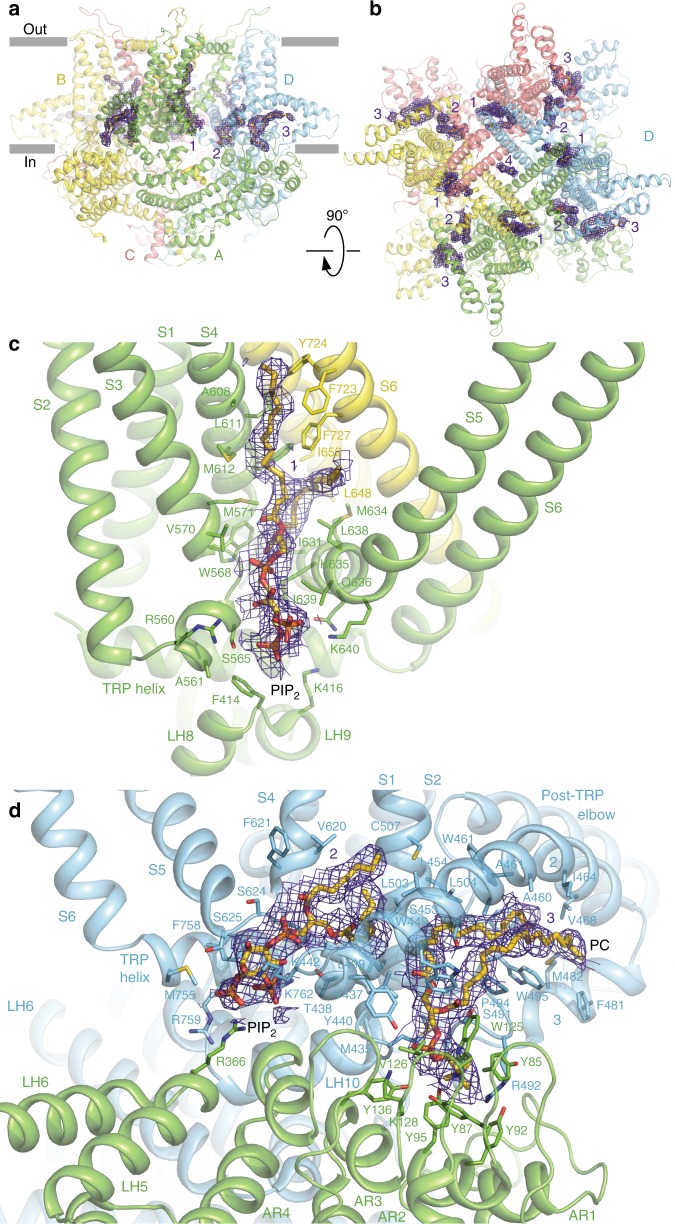
Auxiliary lipids. **a**, **b**, Side (**a**) and top (**b**) views of the crTRP1 tetramer, with each subunit shown in a different color and putative lipid densities 1–4 illustrated by purple mesh. **c**, **d** Expanded views of lipid densities 1 (**c**) and 2–3 (**d**). Molecules of PIP_2_ and PC fitted in densities 1–2 and 3, respectively; the surrounding residues are shown as sticks

We identified twelve (three per subunit) well-resolved, non-protein densities in the crTRP1 cryo-EM map that are buried in the inter-subunit intramembrane interfaces (Fig. 5). Because of the twofold symmetry of crTRP1, these putative lipid densities and corresponding binding sites are slightly different in subunits A and C, when compared to B and D (Supplementary Fig. 4c, d). On the basis of the shapes of the putative lipid densities and the proximity of their apparent head domains to positively charged residues, we have modeled the site 1 and 2 densities as PIP_2_. The location of site 1 is analogous to the vanilloid binding site in TRPV1 (ref. ^32^). Site 1 is formed by the C-terminal portion of S4, the N-terminal portion of S5, the S2-S3 linker, and the S5 and S6 helices from the neighboring subunit and contains a positively charged region that can accommodate the negatively charged PIP_2_ head group.

Site 2, located similarly to the lipid binding sites found or proposed to exist in TRPC3 (ref. ^26^), TRPC4 (ref. ^25^), NOMC^23^ and TRPM8 (ref. ^21^), is strategically placed and could allosterically couple the pore region, the S4–S5 linker, and the N-terminal portion of S1 and the pre-S1 elbow. Like PIP_2_ binding to TRPM8 (ref. ^21^), the negatively charged phosphate groups of PIP_2_ at site 2 are in proximity to the positively charged R366 from the linker domain, K442 from the pre-S1 elbow, and R759 and K762 from the TRP helix. Homologous to R759, a highly conserved lysine in other TRP channels has been shown to be critical for the PIP_2_ binding and channel activity^33^. Supporting the role of site 2 in modulating crTRP1 function, the R759K substitution resulted in slower activation kinetics and a rightward shifted G–V curve^9^. Furthermore, removal of site 2 by deleting the entire pre-S1 elbow resulted in a greatly reduced *P*_o_ and loss of activation by PIP_2_ (Supplementary Fig. 5).

Unique to crTRP1, site 3 is sandwiched between the pre-S1 elbow and the ARD fingers of the neighboring subunit. The head of the site 3 putative lipid, which we modeled as phosphatidylcholine (PC), is surrounded by seven tyrosines, two from the pre-S1 elbow and five from the ARD fingers. The acyl tails of PC fit snugly into a large hydrophobic cavity under the long arcing helix 2 of the pre-S1 elbow. As the pre-S1 elbow deletion eliminates site 3, the altered function of the deletion mutant (Supplementary Fig. 5b–d) suggests that a lipid at site 3 might play both structural and functional roles. We also found a non-protein density in the ion conduction pathway at the level of Y747, in the intracellular mouth of the channel, into which a cholesterol molecule can be roughly fit in different orientations (Supplementary Fig. 4e). Like the other non-protein densities, however, its identity is not unambiguous.

### Ion channel pore

Similar to other tetrameric ion channels, the pore of crTRP1 is lined by the S6 helices and the extended portions of the P-loops. For crTRP1 in GDN, the density for the P-loops was completely invisible, presumably due to their higher flexibility, while for crTRP1 in nanodisc, the purported helical portions of the P-loops were poorly resolved and modeled as poly-alanine helices (Supplementary Fig. 2g, h). The selectivity filter and the region between the pore helix and the S6 helix were not built in the structure of TRPM8 due to a lack of interpretable cryo-EM density, and the region connecting S5 and S6 of PKD1 also could not be built in the structure of the PKD1/PKD2 complex^28,34^. In the case of crTRP1, it is possible that in a lipid bilayer the P-loops are more flexible than those of other TRP channels, however, it is also possible that the disordered P-loops are the product of our experimental methods and are not flexible during, for example, planar bilayer recordings or in a cell membrane. Nevertheless, the P-loops must have an important functional role in crTRP1 as their deletion resulted in substantially impaired gating and reduced single-channel conductance (Supplementary Fig. 5a, c, d). Preceding the helical portions of the crTRP1 re-entrant pore loops, the extracellular turret-forming S5-P-loops contain a disulfide bridge (Supplementary Fig. 6). An analogous disulfide bridge is present in the S5-P-loops of TRPC4, while in the TRPM4 turret, it is incorporated in the P-S6-loop instead. Substitution of the bridge-forming cysteines in TRPC4 results in altered channel function^25^. Similarly, we show that crosslinking of the extracellular turret cysteines could be important for crTRP1 function; the presence of 100 μM dithiothreitol (DTT) drastically reduces the crTRP1 *P*_o_ (Supplementary Fig. 7a, b). The formation of such a disulfide bond could be important for the behavioral responses of *C*. *reinhardtii*. Indeed, it has been shown that thermotaxis in *C*. *reinhardtii* is controlled by redox conditions^35^.

**Fig. 6:**
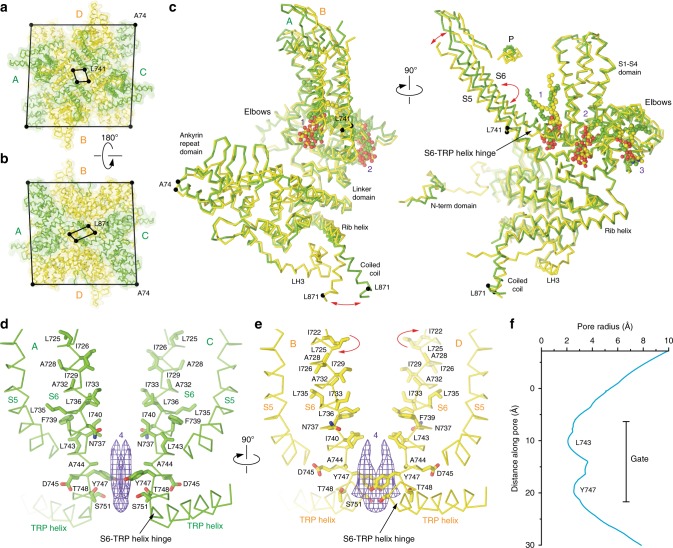
Twofold symmetry and pore. **a**, **b** Extracellular (**a**) and intracellular (**b**) views of the crTRP1 tetramer with pairs of diagonal subunits A/C and B/D colored green and yellow, respectively. Cα atoms of A74, L741, and L871 are shown as dark spheres connected by lines. **c** Superposition of subunits A and B, with lipid molecules shown in space-filling representation. Relative displacements of the S5 and coiled coil helices and rotation of S6 are indicated by red arrows. **d**, **e** Pore-forming regions in subunits A/C (**d**) and B/D (**e**), with S6 residues shown as sticks and putative lipid density 4 illustrated by purple mesh. **f** The pore radius (excluding P-loop region) calculated using HOLE^49^

Of all domains, the ion channel pore reflects the twofold symmetry of crTRP1 most distinctly. Apart from the pore-forming and coiled coil helices, the overall crTRP1 symmetry is close to fourfold (Fig. 6a–c). While deviation from the fourfold symmetry at the coiled coil region is accommodated by a simple swing of the coiled coil helices in subunits A/C relative to subunits B/D, the pore-forming S6 helices in B/D undergo one helical turn (~100°) rotation with respect to the S6 helices in A/C, exposing a different set of residues to the pore. Superposition of subunits A and C onto ~90° rotated B and D subunits show that the C termini of TRP helices in the two subunit pairs overlap (Supplementary Fig. 8). The N-terminal ends of the TRP helices from subunits B and D move up (toward the extracellular side of the protein) and rotate in the plane of the membrane, when compared to subunits A and C. Superposing subunits A and B based on their TRP helices reveals that the linker, pre-S1 and S1–S4 domains do not move much in subunit A relative to subunit B, while S5 and S6 move away from the S1–S4 helices (Supplementary Fig. 8d). Also, the S4–S5 linker moves closer to the TRP helix in subunit B relative to subunit A. Different relative positioning of domains in subunit B is accompanied by rearrangement of the side chains of Q632 and R761 but the overall interaction between the TRP helix and the domains surrounding it does not change very much between subunits. As the TRP helices maintain the same overall conformation in A/C and B/D subunits, the S6 rotation also results in subunit-specific conformations of the S6-TRP helix hinge (Fig. 6d–e, Supplementary Fig. 8b–d). Similar rotation in only two of four pore-forming subunits was observed in the twofold symmetrical structure of TRPV2_RTx-ND_^36^. In TRPV2, however, only the C-terminal portions of S6 underwent ~100° rotation, yielding an α-to-π transition in only two diagonally opposed subunits. In contrast, the S6 helices in all four crTRP1 subunits are entirely α-helical and rotate in their entirety.

The twofold symmetrical arrangement of the pore-lining S6 helices results in different contributions to the S6 bundle crossing gate; the B/D subunits appear to adopt a more closed conformation while the A/C subunits are more open. Indeed, L743 in the B/D S6 helices form the narrowest (6.1 Å) crTRP1 pore constriction (Fig. 6e), which is intermediate in diameter when compared to the constrictions formed by I679 in the TRPV1 pore in the apo (5.3 Å) and capsaicin-bound (7.6 Å) states^32^. In contrast, the narrowest crTRP1 pore constriction contributed by subunits A/C (8.2 Å) is formed by I740 (Fig. 6d) and is intermediate in diameter when compared to the TRPV1 pore constrictions in the capsaicin- and RTX/DkTx-bound (9.3 Å) states^32^. While the minimal radius of the twofold symmetrical crTRP1 pore (Fig. 6f) is large enough for ion permeation, it may represent an intermediate gating state, similar to the state of the twofold symmetrical TRPV3_2-APB_ structure or the TRPV2_RTx-ND_ structure^36,37^. TRPV3, and likely TRPV2, can adopt twofold symmetric structures while transitioning between states that exhibit fourfold symmetry. That twofold symmetrical structures have been determined for distantly related channels indicates that four- to twofold symmetry transitions might be a conserved component of the gating mechanisms of TRP channels and could be more prevalent than currently known. Regardless of its symmetry, the crTRP1 ion conduction pathway is occluded in both the GDN and nanodisc structures, as evidenced by a strong density in the middle of the ion channel pore (Fig. 6d, e). The hydrophobic environment at this narrow, gate-forming region of the pore suggests that the density may belong to a lipid. Given its flat shape, this density might represent differently oriented molecules of cholesterol that plug crTRP1 pore (Supplementary Fig. 4e, f).

### crTRP1 structure and evolution

The distinct pore structure and overall architecture of crTRP1 emphasizes its unique evolutionary niche. crTRP1 has evolved from common ancestors of plants and animals, the most recent of which existed ~1.6 billion years ago^38,39^. Combined with functional and sequence analysis^9^, the structure of crTRP1 expands our understanding of TRP channel diversity and evolution. Compared to its mammalian counterparts^19,20,25–27^, crTRP1 includes domains that remain structurally conserved throughout evolution and include the canonical six-helix transmembrane domain, linker domain, TRP helix, rib helix, and coiled coil domains, exhibiting the close structural homology to the TRPC channels (Fig. 3). The TRP helix is homologous to those of mammalian TRP channels and contains conserved residues in the TRP-box signature sequence “WKFQR”, including two positively charged residues at positions 2 and 5 that have been proposed to be important for PIP_2_ binding (Supplementary Fig. 8a). The highly conserved tryptophan at position 1 is replaced by phenylalanine 758, as was noted previously^9^.

**Fig. 7:**
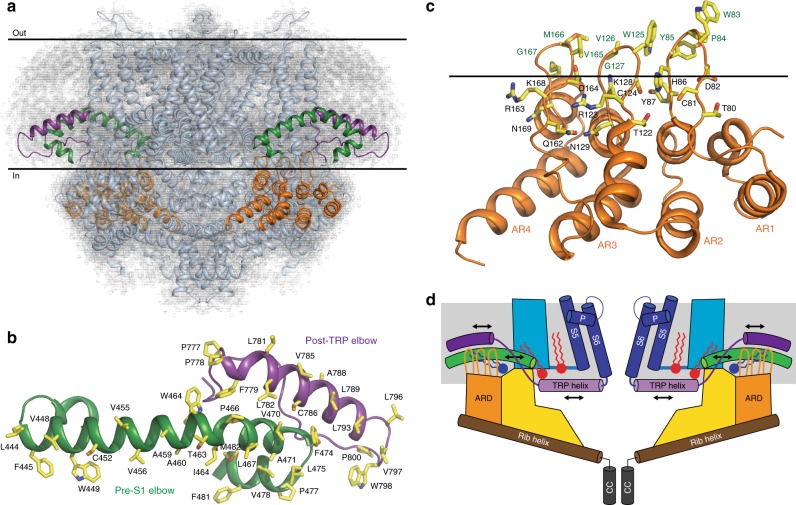
Membrane-linked sensory domains. **a** Side view of crTRP1 tetramer in nanodisc, with two of four pre-S1 elbows colored green, two of four post-TRP elbows colored purple, two of four ARDs colored orange and the rest of the molecule colored cyan. Cryo-EM density is shown as gray mesh; the membrane boundaries are indicated by horizontal lines. **b** Top view of subunit A’s elbows; residues facing the membrane are shown as sticks. **c** Side view of the ARD from subunit D; residues in proximity to the intracellular membrane boundary (horizontal line) are shown as sticks. **d** Cartoon illustrating possible gating mechanism that involves lipids (PIP_2_ in red and PC in dark blue) and that transmits changes in the membrane environment from the ARD and elbow domains through the TRP helices to the S6 gate

Other domains of crTRP1, however, show diverged structural features that are likely to be important for crTRP1 function (Fig. 7). Compared to other TRP channels, the crTRP1 pre-S1 elbow domain evolved to a larger than average size and harbors a unique lipid binding site (site 3), which is located strategically to link the ARD with the pre-S1 and post-TRP elbow domains (Fig. 5). The hydrophobic surfaces of the elbow domains (Fig. 7b) facilitate their submersion into the membrane and suggest their possible role in sensing the composition of the membrane and perhaps the environmental temperature^32^. Linked to the elbow domains by the site 3 lipid-like density, the crTRP1 ARDs adopt a similar overall architecture to the ARDs from the TRPC sub-family, but with uniquely organized fingers (Supplementary Fig. 9). Indeed, the tips of the crTRP1 ARD fingers contain triplets of hydrophobic residues, capable of dipping into the lipid bilayer (Fig. 7c). At the bases of the fingers, the triplets are surrounded by polar and charged residues, capable of interacting with the polar heads of membrane lipids. We speculate that the ARDs function as a part of the crTRP1 scaffold, relative to which the elbow domains move in response to changes in their environment (Fig. 7d). As the TRP helix directly links the post-TRP elbow to the channel gate, the distinct arrangement of the crTRP1 domains might reflect a unique molecular mechanism utilized by *C*. *reinhardtii* to sense temperature and chemical composition of the environment.

## Methods

### Protein expression and purification

The crTRP1 coding sequence was amplified from a plasmid containing *Chlamydomonas Reinhardtii* TRP1 (pTracer_Cr-TRP1), which was a gift from Sebastián Brauchi (Addgene plasmid #64881; http://n2t.net/addgene:64881; RRID: Addgene_64881), using the primers 5′-AAGGGATCCACCATGAAGGTCGCTCCAGCGCCAGCCTCAGGGCAGCCTGG-3′ and 5′-GGCGGCCGCAGAGCCGCGCGGCACCAGGTGCCCCGCAGCGGCGGT-3′ with Q5 polymerase. The amplified product was digested with the *Bam*H1 and *Not*1 restriction enzymes and subcloned into a pEG BacMam vector with a C-terminal thrombin site (LVPRG) followed by eGFP and a streptavidin affinity tag (WSHPQFEK). The crTRP1-PreS1ED (elbow deletion) construct was created from the wild-type crTRP1 BacMam plasmid using the primers 5′-GTCCCACTTCTCGGTGAATATCTGCTG-3′ and 5′-GGCACTGGCCCACTGTACCGGTGGCTGTTGACGCAGTGC-3′. The resulting PCR product was treated with Dpn1 and then T4 Polynucleotide Kinase and was ligated to create a plasmid. The crTRP1-PLD (pore-loop deletion) was created from the wild-type crTRP1 BacMam plasmid using the primers 5′-GGCCTCCTGAAATAGAGCCTCCATGCCCAC-3′ and 5′-GGCACTGGCGAGCAGAAGCGCGTCACCGGGGTCATCTTC -3′. The resulting PCR product was treated as described above for the crTRP1-PreS1ED construct. The resulting plasmids were used for the production of bacmids and baculoviruses as was done previously^40^. The baculovirus was produced in Sf9 cells (Thermo Fisher Scientific, mycoplasma negative, GIBCO #12659017) and was used to transduce HEK 293 S cells (mycoplasma test negative, ATCC #CRL-3022) lacking *N*-acetyl-glucosaminyltransferase I (GnTI^−^) and grown in suspension in Freestyle 293 medium (GIBCO-Life Technologies #12338-018) supplemented with 2% FBS at 37 °C and 5% CO_2_. Approximately 12 h after transduction, 10 mM sodium butyrate was added to the cell suspension and the temperature was reduced to 30 °C for another 38 h before the cells were harvested by centrifugation using a Sorvall Evolution RC Centrifuge (Thermo Scientific) at 5471×*g* for 15 min, washed in phosphate-buffered saline (PBS) pH 8.0 and then pelleted using an Eppendorf Centrifuge 5810 at 3202×*g* for 10 min and frozen at −80 °C. The cell pellet that we used to make the crTRP1-GDN sample was thawed at room temperature and resuspended in lysis buffer containing 20 mM Tris (pH 8.0) and 150 mM NaCl (T-buffer), and supplemented with protease inhibitors (0.8 μM aprotinin, 2 μg/ml leupeptin, 2 μM pepstatin A and 1 mM phenylmethysulfonyl fluoride), and 1 mM β-mercaptoethanol (BME). The cell suspension was sonicated in a Misonix sonicator at level 8 for 3 min total (15 s pulses with intervening 15 s pauses). The slurry was subjected to low-speed centrifugation (3202×*g*) and the resulting supernatant was subjected to higher-speed centrifugation (186,000×*g* for 1 hr) to pellet the protein-containing plasma membranes. The membranes were ground in a dounce homogenizer in the lysis buffer described above and then added to an equal volume of lysis buffer that contained 2% digitonin. The mixture was then stirred at 4 °C for 1 h and subjected again to high-speed centrifugation (186,000×*g* for 1 h) to remove any insoluble material. The supernatant was collected and rocked overnight at 4 °C with streptavidin-linked resin. In the next morning, the resin was washed with size-exclusion chromatography (SEC) buffer containing 20 mM Tris pH 8.0, 150 mM NaCl, 0.01% GDN, and 1 mM BME. The protein was eluted from the resin in SEC buffer containing 2.5 mM d-desthiobiotin and then purified via SEC using a Superose 6 column in the same buffer but lacking d-desthiobiotin. After purification, 10 mM Tris(2-carboxyethyl)phosphine (TCEP) was added to the peak fractions and the protein was concentrated to ~2.0 mg/ml. The protein that was incorporated into nanodiscs was purified similarly and was also first extracted in digitonin and then exchanged into GDN-containing buffer; however, 150 mM NaCl and 20 mM Tris (pH 8.0) in the buffer were replaced with 150 mM KCl, 20 mM Hepes (pH 7.4), 1 µM CaCl_2_, and 20 µM MgCl_2_. Also, we did not add BME or TCEP to the protein that was reconstituted into nanodisc, or that was used for the planar bilayer recordings where we tested the effect of DTT on crTRP1. We mixed the purified protein with MSP2N2 and lipids (POPC and POPE in a 3:1 ratio) in a molar ratio of 1:3:166 (monomer:MSP2N2:lipid). The MSP2N2 was stored in buffer containing 20 mM Tris (pH 8.0) and 150 mM NaCl. The lipids were first dissolved in chloroform that was subsequently evaporated with argon, and then dissolved in buffer containing 150 mM KCl, 1 µM CaCl_2_, 20 mM Hepes (pH 7.4), 20 µM MgCl_2_, and 20% GDN. The nanodisc mixture was rocked at room temperature for 1 h and then moved to 4 °C for rocking overnight. Upon moving the mixture to 4 °C, ~20 mg of pre-wet Bio-beads SM2 were added. The next day, the Bio-beads SM2 were removed and the protein was purified via SEC in buffer containing 150 mM KCl, 20 mM Hepes (pH 7.4), 1 µM CaCl_2_, and 20 µM MgCl_2_, without detergent.

### Cryo-EM sample preparation and data collection

We purchased C-flat (Protochips) CF-1.2/1.3-2Au mesh holey carbon grids and prepared Au/Au grids as described in the literature^41^. Briefly, ~50 nm of gold was deposited onto the grid surface using an Edwards Auto 306 evaporator. The grids were then flipped and placed into a Gatan Solarus (model 950) Advanced Plasma Cleaning System for an Ar/O_2_ treatment (4 min, 50 watts, 35.0 sccm Ar, 11.5 sccm O_2_) to remove carbon. The grids were plasma treated for the second time (H_2_/O_2,_ 20 s, 10 watts, 6.4 sccm H_2_, 27.5 sccm O_2_) prior to 3 µl sample application. A Vitrobot Mark V1 was used to plunge-freeze the crTRP1-nanodiscs grids at 22 °C and 100% humidity using a 2 s blot time, the blot force set to 3, a 20 s wait time. The crTRP1-GDN grids were prepared in the same way, except that Vitrobot was set to 4 °C.

The crTRP1-GDN data were collected on a Titan Krios TEM (FEI) operating at 300 kV equipped with a Gatan K2 Summit direct electron detection (DED) camera using a post-column GIF Quantum energy filter. 2342 micrographs were collected using Leginon^42^ with a pixel size of 1.06 Å in a counting mode across the defocus range of −1.0 to −3.0 µm. The total dose, ~61 e^−^ Å^−2^, was attained by using a dose rate of ~8.0 e^−^ pixel^−1^ s^−1^ across 40 frames for 8 s total exposure time.

The crTRP1-nanodisc data were collected on a Tecnai F30 Polara operating at 300 kV equipped with a Gatan K3 DED camera. 6272 micrographs were collected using Leginon in a counting mode with the pixel size of 0.95 Å across the defocus range of −1.0 to −3.0 µm. The total dose, ~71 e^−^ Å^−2^, was attained either by using the dose rate of ~16 e^− ^pixel^−1^ s^−1^ across 40 frames for 4 s total exposure time or by using the dose rate of ~8 e^− ^pixel^−1^ s^−1^ for 8 s total exposure time.

### Image processing

All data was processed using Relion 3.0 (ref. ^43^). The crTRP1-nanodiscs dataset was processed as follows. Initially, ~2000 particles were manually picked to generate 2D classes that were subsequently used to automatically pick 1,714,216 particles. The particle images were subjected to 2D classification and then 3D classification using an ab-initio model as a reference. Two classes, comprising ~550,000 particles, were combined and refined with C1 and C2 symmetry, at which point it was determined that the channel exhibited the overall C2 symmetry. Nevertheless, because of structural ambiguity in the upper-pore region, the C1 reconstruction was used as a reference for the next round of 3D classification, which was also done without imposing symmetry. Five classes were chosen, combined and refined with C2 symmetry, and post-processed to generate the final ~3.5 Å reconstruction.

The crTRP1-GDN dataset was processed in a similar manner to that described above and the reported resolutions were estimated using the Fourier shell correlation (FSC) = 0.143 criterion. The local resolutions were estimated with unfiltered half maps using ResMap^44^ and EM density visualization was done in UCSF Chimera^45^.

### Model building

The data was of sufficient quality to build the structures de novo. The models were tested for overfitting (Supplementary Figs. 1 and 2) by shifting their coordinates by 0.5 Å (using shake) in PHENIX^46^, refining each shaken map against a corresponding unfiltered half map, and generating densities from the resulting models in Chimera^45^. FSC was calculated between the densities generated in Chimera and both unfiltered half maps and the sum maps, using EMAN2^47^. The local resolutions in our reconstructions were estimated using ResMap^44^. Structures were visualized and figures were prepared in Pymol^46^.

### Planar lipid bilayer measurements

Planar lipid bilayers measurements were performed as described previously^48^. Briefly, planar lipid bilayers were formed from a solution of synthetic 1-palmitoyl-2-oleoyl-sn-glycero-3-phospho-(1′-rac-glycerol) (POPG), 1-palmitoyl-2-oleoyl-glycero-3-phosphocoline (POPC) and 1-palmitoyl-2-oleoyl-glycero-3-phosphoethanolamine (POPE; Avanti Polar Lipids) at a 3:1:1 ratio in *n*-decane (Sigma-Aldrich). The solution was used to paint a bilayer in an aperture of ∼150 µm diameter in a Delrin cup (Warner Instruments) between symmetric aqueous bathing solutions of 150 mM KCl, 0.02 mM MgCl_2_, 1 μM CaCl_2_, and 20 mM HEPES (pH 7.2). Unless specified otherwise, all experiments were performed in the presence of 2.5 μM 1-(1,2R-dioctanoylphosphatidyl)inositol-4,5-bisphosphate, trisodium salt (DiC_8_-PIP_2_, Cayman Chemical) added to both compartments. To assess the effect of a reducing agent on crTRP1-mediated currents, after obtaining heat-induced crTRP1 activity, 100 μM DTT was added to both compartments of the bilayer, and the channel activity was then continuously recorded for up to 5–6 h. All reagents (Sigma-Aldrich) were ultrapure (>99%). Bilayer capacitances were in the range of 50–75 pF.

After the bilayers had formed, the micellar solution of crTRP1 protein (0.02 μg/ml) was added by painting. Unitary currents were recorded using the Axopatch 200B patch-clamp amplifier (Molecular Devices). The *trans* solution (command voltage side) was connected to the CV 201A head-stage input, while the *cis* solution was held at a virtual ground via a pair of matched Ag-AgCl electrodes. Currents through the voltage-clamped bilayers (background conductance, <1 pS) were filtered at the amplifier output (low pass, −3 dB at 10 kHz, 8-pole Bessel response). Data were filtered at 100 Hz through an 8-pole Bessel filter (950 TAF; Frequency Devices) and digitized at 1 kHz with an analog-to-digital converter Digidata 1322A controlled by pClamp10.3 software (Molecular Devices). Single-channel conductance events, all-points histograms, open probability (*P*_o_), and other parameters were identified and analyzed with Clampfit10.3 software (Molecular Devices). The experiments were performed in the temperature range from 22 to 30 °C. For the temperature-dependence measurements, the bilayer recording chamber was fitted onto a conductive stage containing a pyroelectric heater/cooler that was controlled by a temperature controller (CL-100; Warner Instruments). Deionized water was circulated through the stage and pumped into the system to remove the generated heat. The temperature of the bath was constantly monitored using a thermoelectric device in the cis chamber (the ground side) and was reliably controlled within ±0.5 °C. The temperature coefficients (*Q*_10_) for *P*_o_ were calculated using Eq. 1:

1 $$Q_{10} = \left( {\frac{{X_2}}{{X_1}}} \right)^{\frac{{10}}{{T_2 - T_1}}}$$

where *X*_1_ and *X*_2_ are *P*_o_ values obtained at *T*_1_ and *T*_2_ temperatures measured in Kelvins. Statistical analysis was performed using Origin 9.0 (Microcal Software Inc.). Statistical significance was calculated using One-way ANOVA followed by Fisher’s least significant difference test. All data are presented as mean ± SEM.

### Reporting summary

Further information on research design is available in the Nature Research Reporting Summary linked to this article.

## Supplementary information

Supplementary Information

Reporting Summary

Source Data

## Data Availability

Cryo-EM density maps have been deposited to the Electron Microscopy Data Bank (EMDB) under accession numbers EMD-20499 for crTRP1-nanodiscs and EMD-20498 for crTRP1-GDN. Model coordinates have been deposited to the Protein Data Bank (PDB) under accession numbers 6PW5 for crTRP1-nanodiscs and 6PW4 for crTRP1-GDN. The source data underlying Fig. 1b, d, f, h and Supplementary Figs. 5c, d and 7b are provided as a Source Data file. All other data are available from the corresponding authors upon request.
