# Supplementary material for: Structure of the thermo-sensitive TRP channel TRP1 from the alga Chlamydomonas reinhardtii
Source: Nat Commun. 2019 Sep 13;10:4180. doi: 10.1038/s41467-019-12121-9 (PMC6744473; doi:10.1038/s41467-019-12121-9)
Supplement: Supplementary file 1 — Supplementary Information [file 41467_2019_12121_MOESM1_ESM.pdf]

Supplementary Information

Structure of the Thermo-Sensitive TRP Channel TRP1 from the Alga *Chlamydomonas Reinhardtii*

McGoldrick et al.

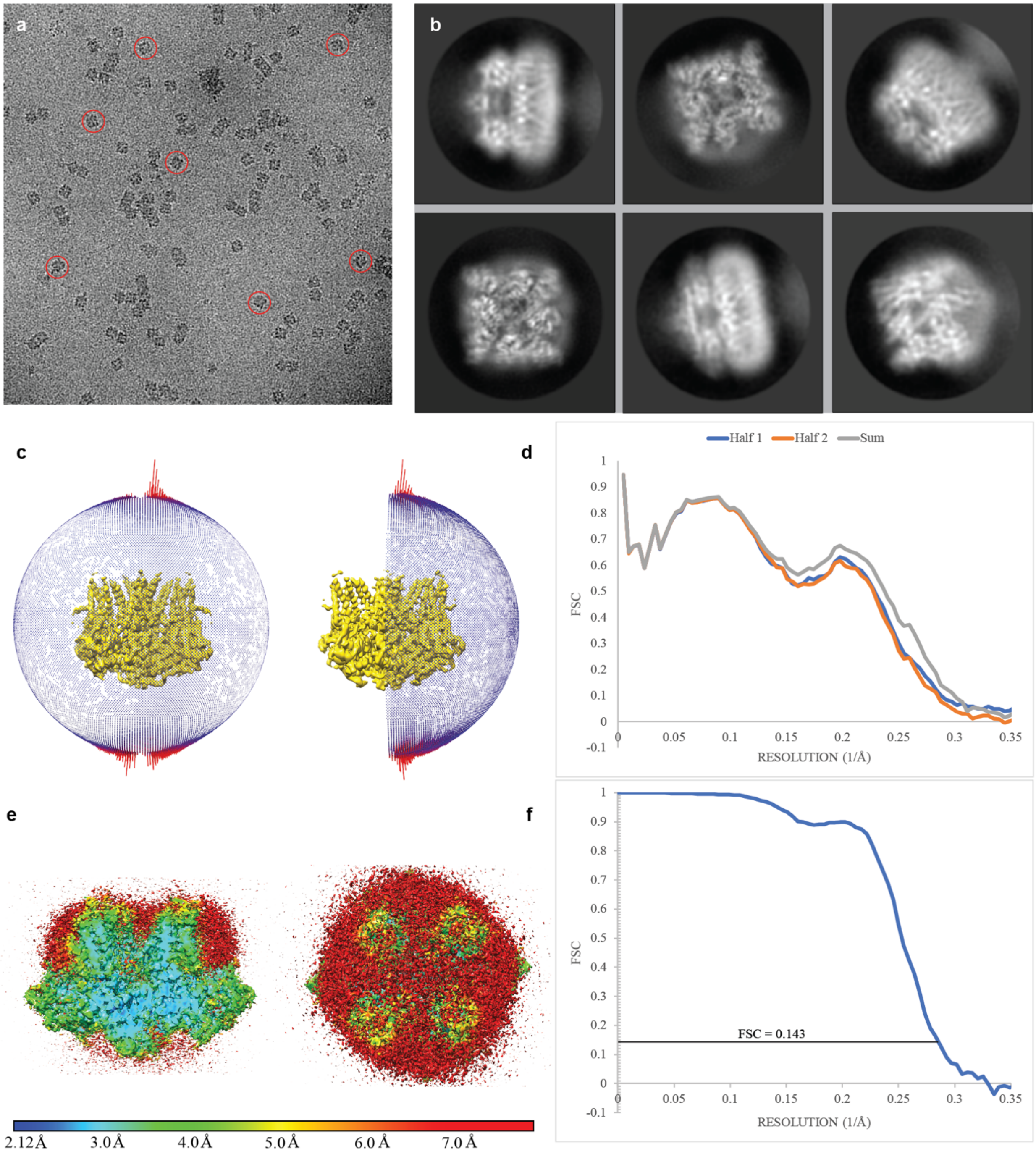

Supplementary Figure 1. Cryo-EM overview of crTRP1 in GDN detergent. a, Micrograph of crTRP1 taken using a Gatan K2 DED with example particles circled in red. **b**, Reference free 2D class averages of crTRP1 in different orientations. **c**, Euler angle distribution of particles contributing to the final reconstruction; larger red cylinders represent orientations comprising more particles. **d**, FSC curves calculated between two unfiltered half-maps and the final map and a model whose coordinates were randomized and refined against only half map 1. **e**, Local resolution predicted by ResMap. **f**, FSC curve calculated between half maps.

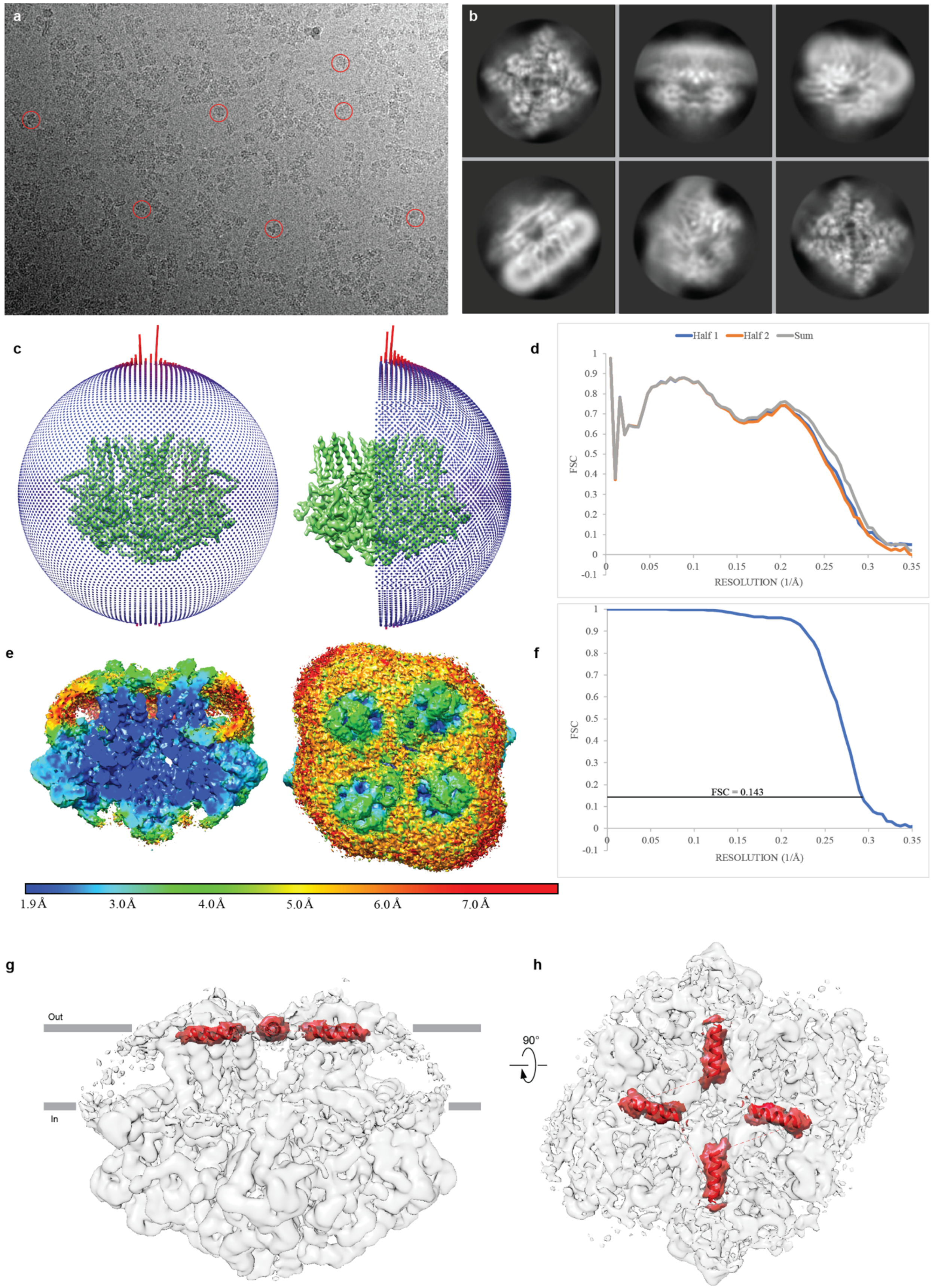
a
b
c
d
Half 1
Half 2
Sum
FSC
RESOLUTION (1/Å)
e
1.9 Å
3.0 Å
4.0 Å
5.0 Å
6.0 Å
7.0 Å
f
FSC = 0.143
FSC
RESOLUTION (1/Å)
g
Out
In
h
90°

Supplementary Figure 2. Cryo-EM overview of crTRP1 in nanodiscs. **a**, Micrograph of crTRP1 taken using a Gatan K3 DED with example particles circled in red. **b**, Reference free 2D class averages of crTRP1 in different orientations. **c**, Euler angle distribution of particles contributing to the final reconstruction; larger red cylinders represent orientations comprising more particles. **d**, FSC curves calculated between two unfiltered half-maps and the final map and a model whose coordinates were randomized and refined against only half map 1. **e**, Local resolution predicted by ResMap. **f**, FSC curve calculated between half maps. **g-h**, Close-up views of the cryo-EM map illustrating densities for the P-loop helical portions modelled as poly-alanine α-helices.

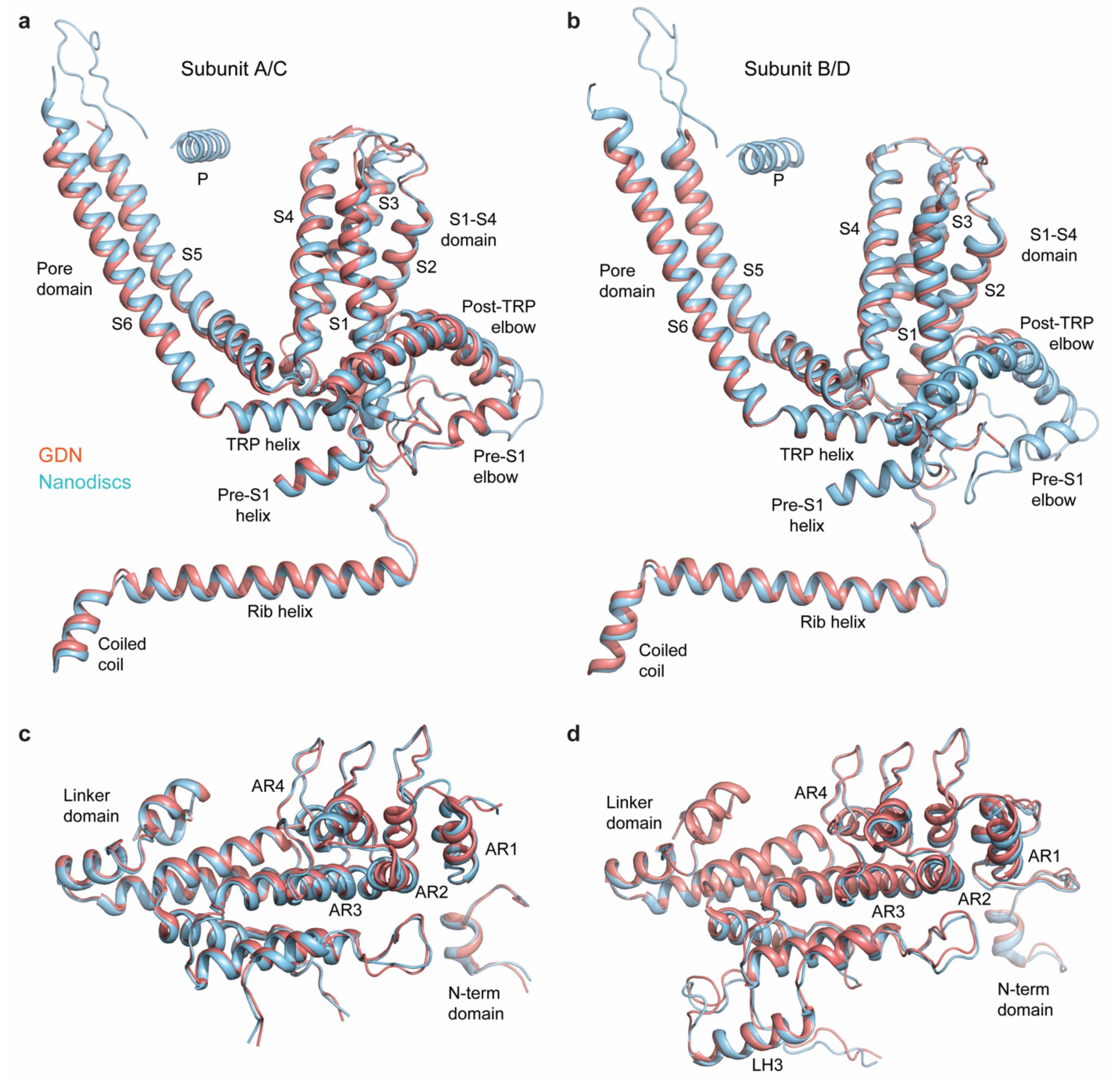

Supplementary Figure 3. Comparison of crTRP1 structures in GDN and nanodiscs. a-b, Structural superposition of the C-terminal portions of subunits A/C (**a**) and B/D (**b**) from crTRP1 structures in GDN (pink) and nanodisc (cyan). **c-d**, Similarly colored structural superposition of the N-terminal portions of subunits A/C (**c**) and B/D (**d**).

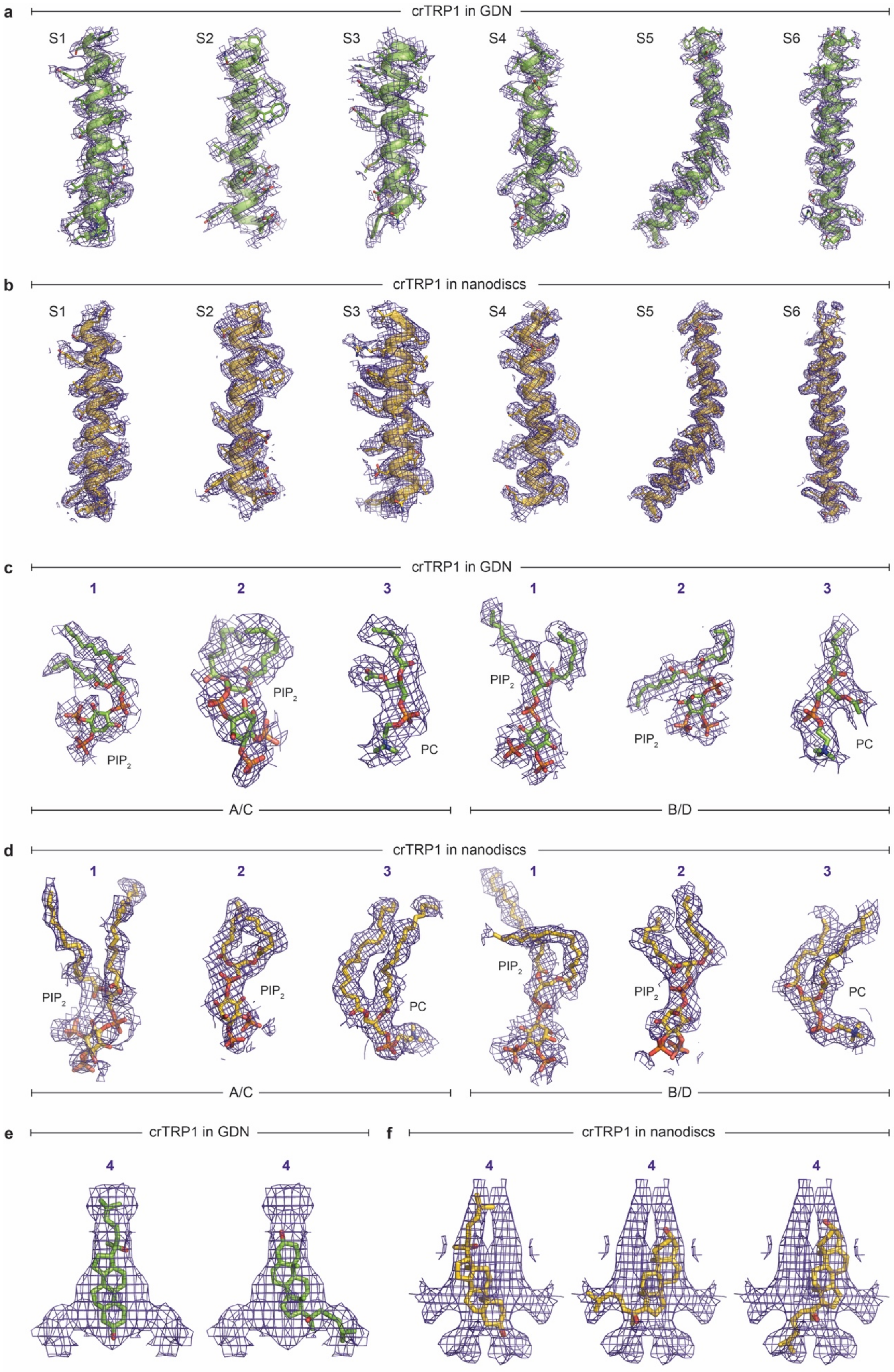
a
crTRP1 in GDN
S1
S2
S3
S4
S5
S6
b
crTRP1 in nanodiscs
S1
S2
S3
S4
S5
S6
c
crTRP1 in GDN
1
2
3
1
2
3
PIP2
PIP2
PC
PIP2
PIP2
PC
A/C
B/D
d
crTRP1 in nanodiscs
1
2
3
1
2
3
PIP2
PIP2
PC
PIP2
PIP2
PC
A/C
B/D
e
crTRP1 in GDN
4
4
f
crTRP1 in nanodiscs
4
4
4

Supplementary Figure 4. Cryo-EM density. a-b, Transmembrane domain fragments of crTRP1 structures (subunit A) in GDN (**a**) and nanodisc (**b**), with the protein colored green (**a**) and yellow (**b**) and the corresponding cryo-EM density shown as purple mesh. **c-d**, Cryo-EM density of putative auxiliary lipids 1-3 fitted with molecules of PIP_2 (1-2) and PC (3), shown for the diagonal subunit pairs A/C and B/D of the crTRP1 structures in GDN (**a**) and nanodisc (**b**). **e-f**, Cryo-EM density of the putative central pore lipid 4 for the crTRP1 structures in GDN (**e**) and nanodisc (**f**), and fitted with cholesterol molecules in different orientations.

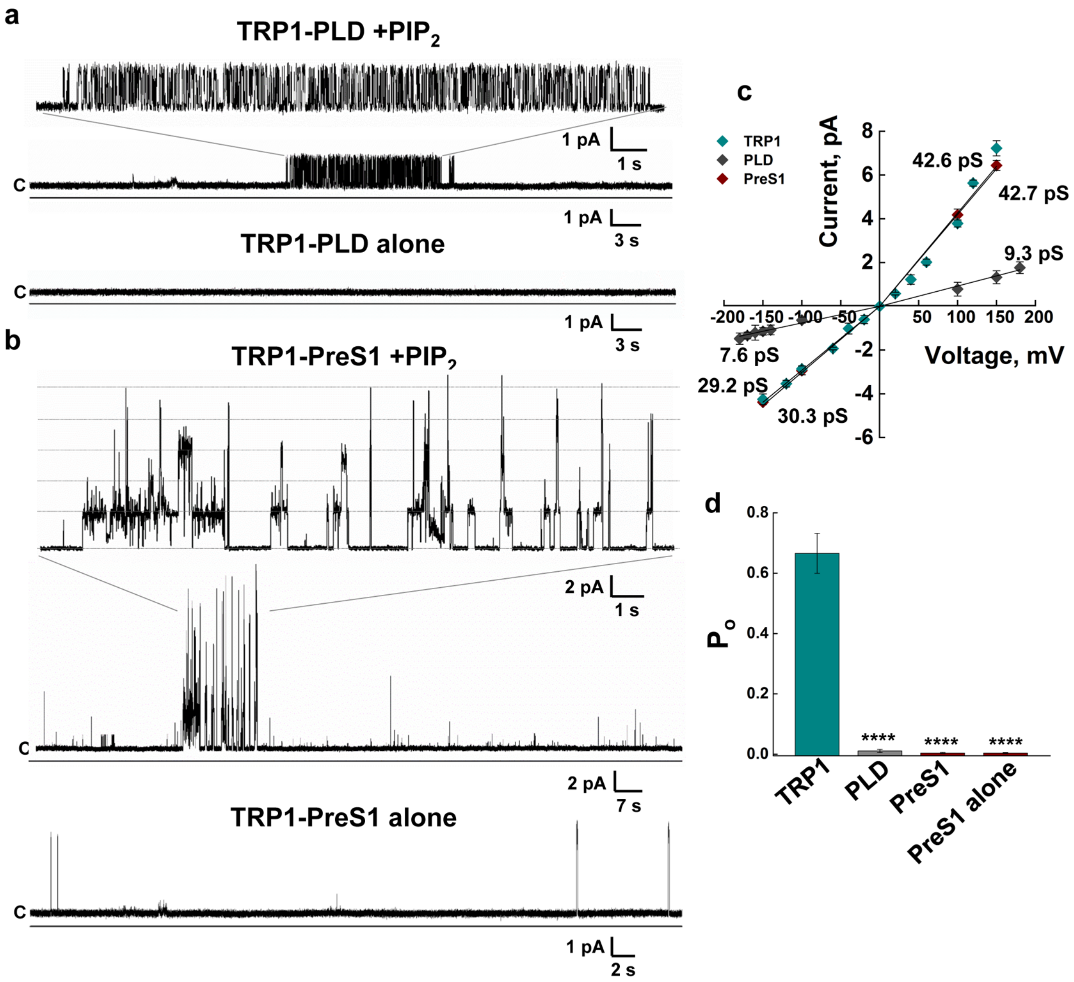

Supplementary Figure 5. Functional characterization of crTRP1 mutants. **a**, Representative planar lipid bilayer recordings of crTRP1-PLD obtained at 28 ºC and +100 mV in the presence (upper traces) or absence (lower traces) of 2.5 μM PIP_2 (n = 5; NE = 8,407). **b**, Representative current traces obtained for TRP1-PreS1ED (elbow deletion) in the presence (upper traces) or absence (lower traces) of 2.5 μM PIP_2 (n = 8; NE = 3,197). **c,** I-V curves for wild type crTRP1, PLD (pore-loop deletion), and PreS1ED mutants obtained at 28ºC (n = 27; NE = 167,158) **d,** Average values of P_o measured at +100 mV for wild type and mutants (n = 16 independent experiments, NE = 78,347). The differences between P_o values were calculated using One-Way ANOVA, with p = 2.4 x 10^{-6} for the control vs. PLD, p = 4.9 x 10^{-7} for the control vs. PreS1ED, and p = 1.2 x 10^{-7} for the control vs. PreS1ED in the absence of PIP_2. Error bars show SEM. Source data are provided as a Source Data file.

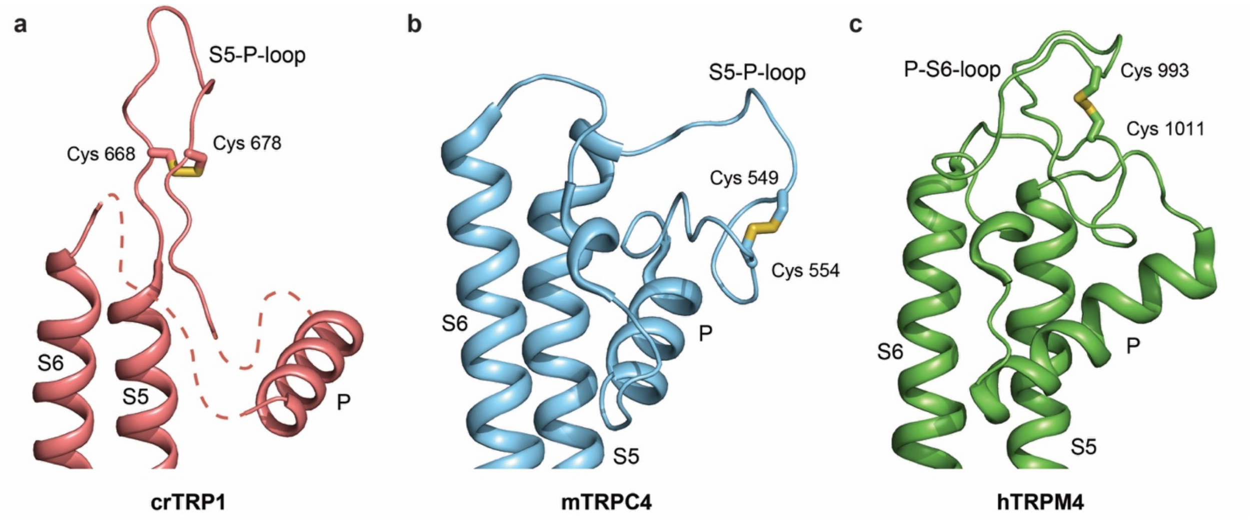

Supplementary Figure 6. Disulfide bridges in the extracellular turrets of different TRP channels. a-c, Views of the extracellular turret-forming region encompassing the P-loop in a single subunit of crTRP1 (**a**), mouse TRPC4 (**b**, PDB ID: 5Z96) and human TRPM4 (**c**, PDB ID: 6BQR). Crosslinked cysteines are shown as sticks. Unresolved regions of crTRP1 are shown as dashed lines.

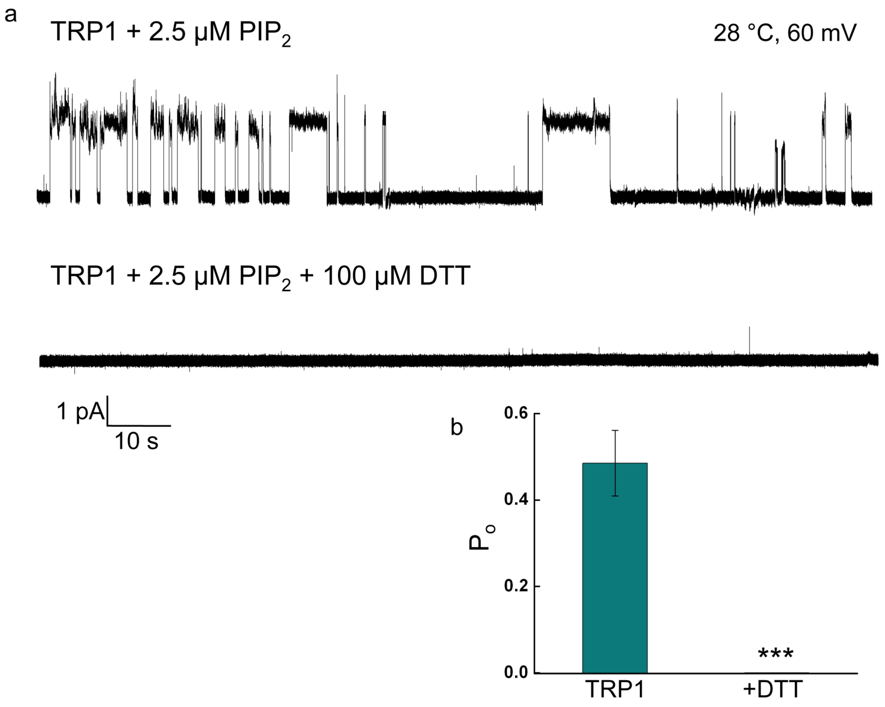

Supplementary Figure 7. A reducing agent disrupts crTRP1 channel gating. a, Representative single channel recordings of TRP1 in the presence of 2.5 µM PIP_2 at 28 ºC, held at 60 mV, before (top traces; P_o 0.49 ± 0.076) and after the addition of 100 µM DTT (bottom traces; P_o 2.5 x 10^{-5} ± 2.5 x 10^{-5}). **b**, Open probability (P_o) of TRP1 in the absence and presence of 100 µM DTT, p = 6.9 x 10^{-4} (n = 8, number of analyzed events = 1,264). Errors are SEM. Source data are provided as a Source Data file.

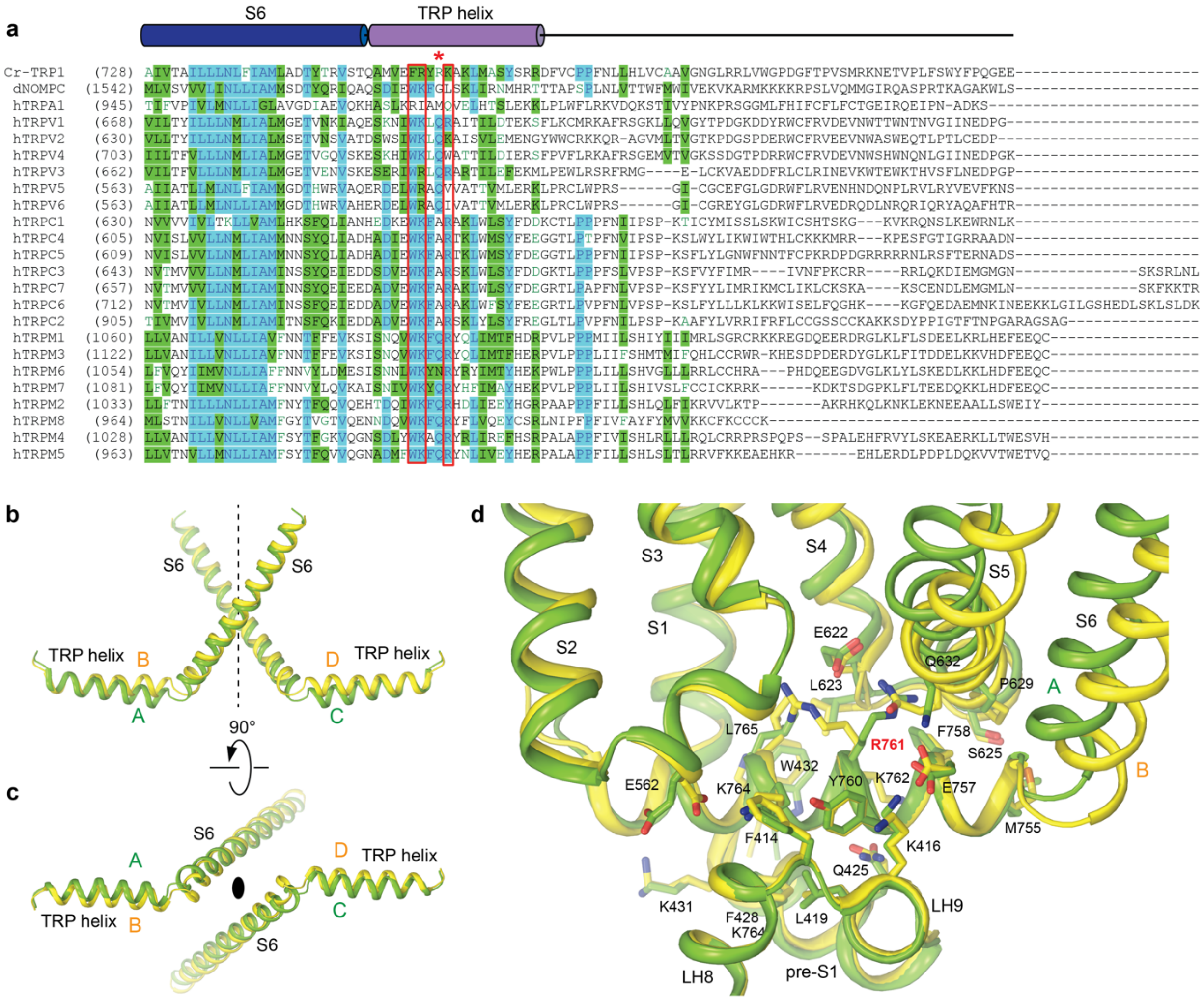

Supplementary Figure 8. Sequence and Structure Analysis of the TRP domain of crTRP1. a, Sequence alignment of the TRP domains from crTRP1 and other TRP channels. Three residues of the "WKFQR" TRP-box signature sequence are boxed. The tryptophan at position 1 is replaced by a phenylalanine and the positive charge is conserved at positions 2 and 5. Arginine R761, which is in different conformations in the two diagonal pairs of crTRP1 subunits (**d**), is indicated by a red star symbol. **b-d** Superposition of the S6 and TRP helices from diagonal pairs of subunits of a crTRP1 tetramer (**b**, **c**) and a view of the superposed TRP domains in subunits A and B with the surrounding residues (**d**).

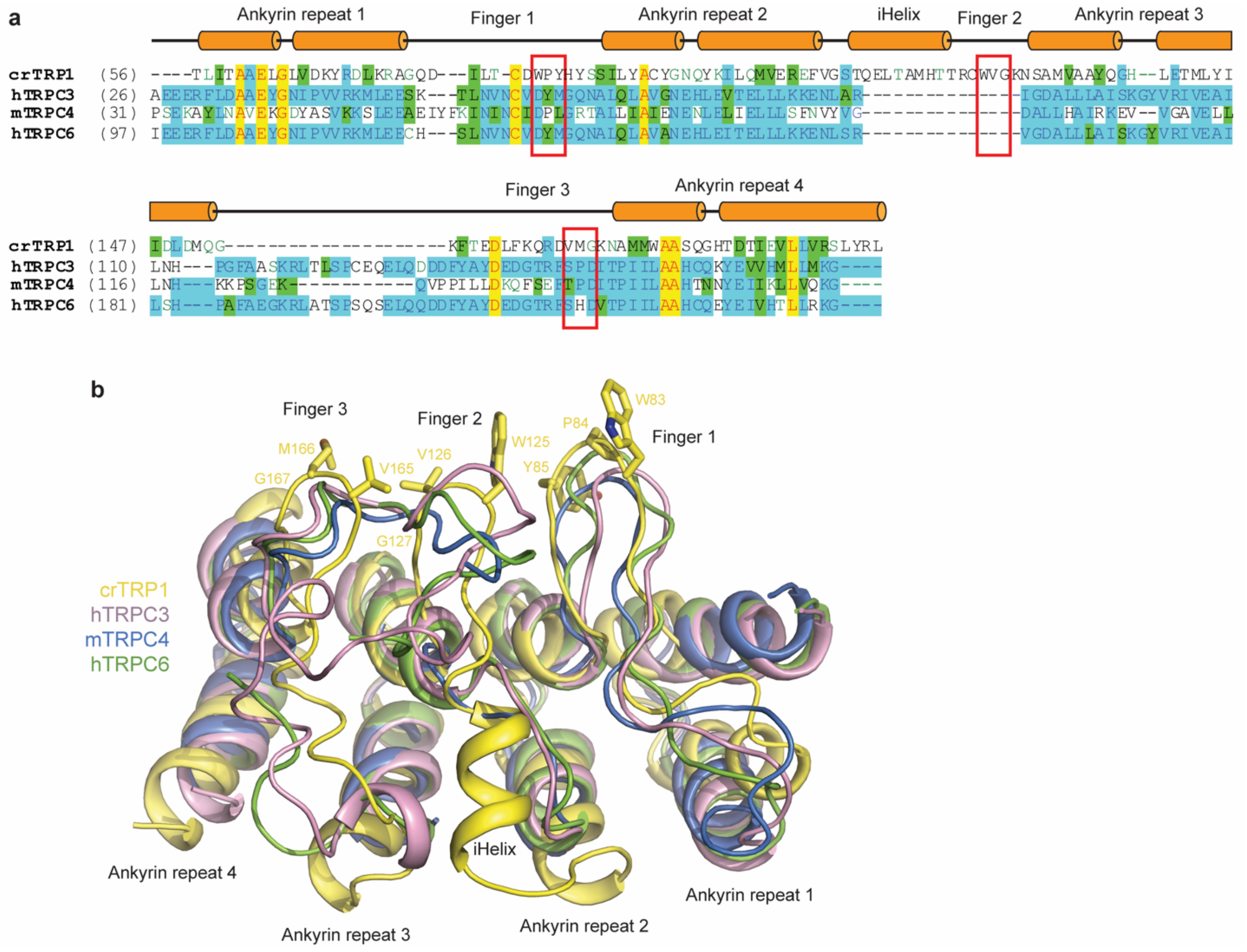

Supplementary Figure 9. Comparison of the ankyrin repeat domains in crTRP1 and TRPC channels. a, Structure-based sequence alignment of the ARDs in crTRP1 and human TRPC3, 4 and 6. Cylinders above the alignment indicate α-helical regions, while red boxes indicate the ARD finger-tip regions. **b**, Structural superposition of the ARDs from crTRP1 (yellow), human TRPC3 (purple, PDB ID: 6CUD), mouse TRPC4 (blue, PDB ID: 5Z96) and human TRPC6 (green, PDB ID: 5YX9).

Supplementary Table 1. Cryo-EM data collection, refinement and validation statistics

	crTRP1-GDN (EMDB-20498) (PDB 6PW4)	crTRP1-nanodisc (EMDB-20499) (PDB 6PW5)
**Data collection and processing**		
Magnification	130,000x	39,000x
Voltage (kV)	300	300
Electron exposure (e–/Å^2)	~61	~71
Defocus range (μm)	-1.0 to -3.0	-1.5 to -3.5
Pixel size (Å)	1.06	0.95
Symmetry imposed	C2	C2
Initial particle images (no.)	310,882	1,714,216
Final particle images (no.)	63,018	255,973
Map resolution (Å)	3.53	3.45
FSC threshold	0.143	0.143
Map resolution range (Å)	2.12-5	1.9-4
**Refinement**		
Initial model used (PDB code)	This Study	This Study
Model resolution (Å)	3.53	3.45
FSC threshold	0.143	0.143
Model resolution range (Å)	2.12-5	1.9-4
Map sharpening *B* factor (Å^2)	-116	-188
Model composition		
Non-hydrogen atoms	22,348	24,740
Protein residues	2742	3032
Ligands	10	12
B factors (Å^2)		
Protein	50.09	42.43
Ligand	59.91	50.83
R.m.s. deviations		
Bond lengths (Å)	0.007	0.013
Bond angles (°)	1.081	1.341
Validation		
MolProbity score	1.87	1.98
Clashscore	5.63	4.38
Poor rotamers (%)	0.38	1.61
Ramachandran plot		
Favored (%)	89.27	87.62
Allowed (%)	10.66	11.62
Disallowed (%)	0.07	0.77
